# *sedimix*: a workflow for the analysis of hominin nuclear DNA sequences from sediments

**DOI:** 10.1093/bioinformatics/btag004

**Published:** 2026-01-09

**Authors:** Jierui Xu, Elena I Zavala, Priya Moorjani

**Affiliations:** Department of Molecular and Cell Biology, University of California, Berkeley, Berkeley, CA, 94720, United States; Department of Molecular and Cell Biology, University of California, Berkeley, Berkeley, CA, 94720, United States; Department of Molecular and Cell Biology, University of California, Berkeley, Berkeley, CA, 94720, United States; Center for Computational Biology, University of California, Berkeley, Berkeley, CA, 94720, United States

## Abstract

**Summary:**

Sediment DNA—the recovery of genetic material from archaeological sediments—is an exciting new frontier in ancient DNA research, offering the potential to study individuals at a given archaeological site without destructive sampling. In recent years, several studies have demonstrated the promise of this approach by extracting hominin DNA from prehistoric sediments, including those dating back to the Middle or Late Pleistocene. However, a lack of open-source workflows for analysis of hominin sediment DNA samples poses a challenge for data processing and reproducibility of findings across studies. Here, we introduce a snakemake workflow, *sedimix*, for processing genomic sequences from archaeological sediment DNA samples to identify hominin sequences and generate relevant summary statistics to assess the reliability of the pipeline. By performing simulations and comparing our results to two published studies with human DNA from ∼25,000 years ago (including shotgun data from a sediment sample and capture data from touch DNA recovered from a deer tooth pendant) we demonstrate that *sedimix* yields accurate and reliable inferences. *sedimix* offers a reliable and adaptable framework to aid in the analysis of sediment DNA datasets and improve reproducibility across studies.

**Availability and implementation:**

*sedimix* is available as an open-source software with the associated code, example data, and user manual with installation instructions available at https://github.com/jierui-cell/sedimix. A permanent archived version of this release is available via Zenodo: https://doi.org/10.5281/zenodo.17244854.

## 1 Introduction

The ability to recover ancient hominin DNA from sediments (Slon *et al.* 2017, Gelabert *et al.* 2021, Vernot *et al.* 2021, Zavala *et al.* 2021) and other non-hominin skeletal remains (Essel *et al.* 2023) has revolutionized the study of human evolution. Sediment DNA has the potential to fill in gaps left by a sparse skeletal record and allow for more continuous explorations of human evolution through time and space. However, analyzing sediment DNA is inherently complex, as DNA from many diverse taxa (ancient and modern) are expected to be present, compounding the existing challenges of fragmented, deaminated sequences and low endogenous content inherent with ancient DNA (Briggs *et al.* 2007, Allentoft *et al.* 2012, Sawyer *et al.* 2012, Dabney *et al.* 2013). The identification of authentic ancient hominin sequences is, thus, challenging and available pipelines for genetic analysis from metagenomic sources (e.g. HOPS and MALT) are not optimized and therefore likely lack sensitivity for the detection of low-abundance ancient hominin DNA amidst complex environmental backgrounds. To our knowledge, there are no open-source workflows available for processing sediment sequencing data for the identification of hominin sequences, hindering the analysis and reproducibility of findings across studies.

Sediment DNA studies (Gelabert *et al.* 2021, Vernot *et al.* 2021) utilize two primary approaches for the identification of hominin DNA sequences from metagenomic datasets. The first, composition-based methods, assign taxonomic labels to sequencing reads by comparing the data to existing databases of sequences from diverse species. These approaches use a k-mer based taxonomic classification using tools such as Kraken2 (Wood *et al.* 2019) and Centrifuge (Kim *et al.* 2016). Another approach, alignment-based methods, map the sequencing reads to multiple selected reference genomes. For alignment-based classification, both general-purpose aligners such as bwa (Li and Durbin 2009) and Bowtie2 (Langmead and Salzberg 2012) and specialized aligners suited for metagenomic datasets such as MALT (Herbig *et al.* 2016) are used. For analysis of sediment DNA, it is typical to combine both alignment and classification steps, however, the order, the choice of methods and filtering steps differ and can impact the sensitivity and accuracy of identifying hominin DNA, as well as computational tractability of the analyses.

Here, we present a snakemake workflow, referred to as *sedimix*, for processing sediment DNA data to identify hominin DNA sequences. As input, *sedimix* takes raw sequencing data (fastq files) and returns an alignment file (bam) with sequences inferred as deriving from hominin or other sources. It also outputs a table of summary statistics per sample including the number of mapped sequences, average duplication rates, percent deamination, and percent hominin derived sequences. The user also has the option to generate a fastq file of all non-hominin sequences identified that may be useful for other analyses. We perform simulations to test the sensitivity, precision, and computational requirements of different steps of the workflow and apply *sedimix* to published datasets to demonstrate its robustness. *sedimix* offers a reliable and adaptable pipeline for the analysis of nuclear hominin sequences from archaeological sediment DNA datasets.

## 2 Materials and methods

**Figure 1:**
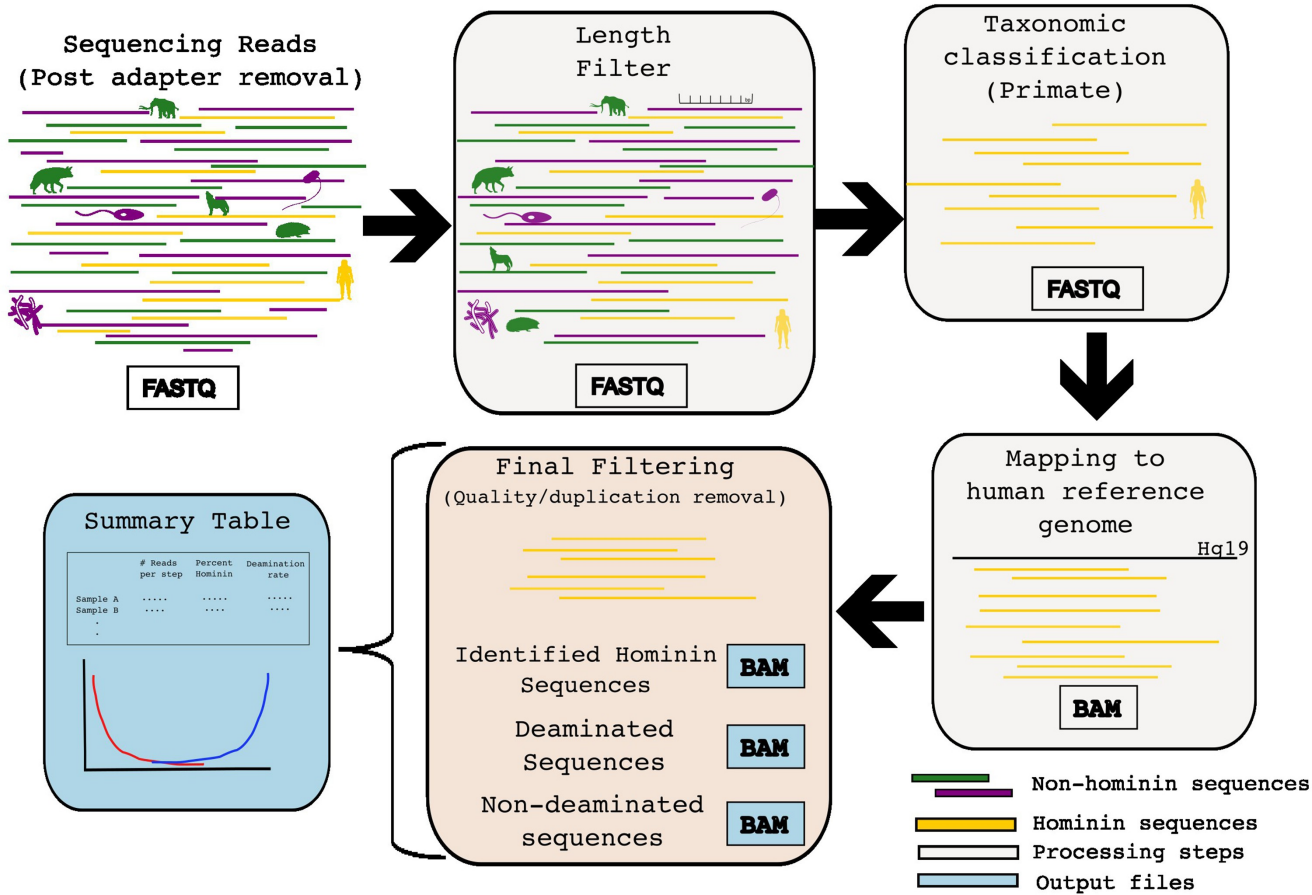
Overview of the different steps in the *sedimix* workflow. The input is a fastq file post adapter removal, which is then filtered based on read length. Next classification is performed and only reads identified as primate are retained. These reads are then mapped to the human reference genome followed by duplicate removal and filtering based on mapping quality. The output are three different bam files and a table with summary statistics per fastq file processed. Silhouettes were obtained from PhyloPic (https://www.phylopic.org) and are available for reuse under Creative Commons licenses.

Our workflow, *sedimix*, can be broken down into four main steps: filtering, classification, mapping, and generating summary statistics (Fig. 1). In order to run the workflow the user must input (1) the fastq file(s) containing the raw sequencing reads, (2) the human reference genome sequence (in fasta format), and (3) a list of parameters to use (see *sedimix* github for details, https://github.com/jierui-cell/sedimix). The latter contains a list of parameters such as mapping quality and read length filters, identification of non-hominin reads, and selected classifier method (Kraken2 or Centrifuge). Users may wish to adjust read length and classifier method depending on expectations of read length distributions for their endogenous DNA, their existing classifiers, databases and/or the overall precision and specificity expectations given their data composition. In addition, the user can add an optional bed file containing a list of positions matching a SNP panel for capture array or ascertainment scheme of interest [in the same coordinates as the human reference genome used in input (2)]. In this case, the reference genome will be modified at the specified positions to a non-reference or third allele (neither ancestral nor derived) in order to reduce reference bias as proposed in earlier studies (Günther and Nettelblad 2019, Vernot *et al.* 2021). The required software and databases for running *sedimix* are listed in Table 1, available as supplementary data at *Bioinformatics* online.

The *sedimix* workflow starts with a filtering step that involves removal of reads that are below a given length cut-off as specified in the parameter list (default: 30 base pairs). Next, the filtered reads are input into the specified classifier (default: Centrifuge, see Section 3, available as supplementary data at *Bioinformatics* online for comparison of classifiers) and all the reads identified as “primate” are extracted. While our goal is to identify hominin reads, we retain all sequences classified as “primate” to maximize sensitivity at this step. Users can optionally extract reads classified to non-primate lineages for downstream analysis.

The third step is to map the primate reads to the user provided human reference genome using bwa aln with optimal parameters for ancient DNA specimens [options: -n 0.01 -o 2 -l 16500 (Schubert *et al.* 2012)]. Following mapping, sequences below a minimum mapping quality score (default: 25) and duplicates identified using samtools markdup (Bonfield *et al.* 2021, Danecek *et al.* 2021) are removed. If a SNP panel was provided by the user, we use bedtools intersect (Quinlan and Hall 2010) to subset the sequences that overlap the target SNP positions. This output bam file contains filtered sequences that map to the human reference genome. To support quality control analysis of these sequences, we split the output file into two separate files: one containing deaminated sequences which are a hallmark of authentic ancient DNA sequences (with a C-to-T or G-to-A substitutions in the first or last 3 bases) and another one containing non-deaminated sequences. The last step in the workflow is to generate a report with summary statistics about the output bam file. The report contains the number of reads/sequences remaining after each filtering step, the number of deaminated sequences, the inferred percentage of deamination based on mapDamage (Jónsson *et al.* 2013), and an estimate of the percentage of hominin DNA within the primate sequences (see Section 2.4, Table 2, available as supplementary data at *Bioinformatics* online for detailed descriptions).

## 3 Results

To evaluate the performance of *sedimix*, we simulated three datasets using an ancient DNA simulator, gargammel (Renaud *et al.* 2017). The simulated datasets had different compositions of sequencing reads as follows: (i) reads were composed of 90% modern human [Individual NA12778 from 1000 genomes (1000 Genomes Project Consortium *et al.* 2015)] and 10% Neanderthal [Altai (Prüfer *et al.* 2014)]; (ii) 40% modern human, 10% Neanderthal and 50% bacteria [composition from Seguin-Orlando *et al.* (2014)]; and (iii) 10% modern human, 1% Neanderthal, 44.5% mammals and 44.5% bacteria (Table 3, available as supplementary data at *Bioinformatics* online). These datasets were selected to test differences in sensitivity and precision between the identification of hominin DNA in general (Sections 3.1–4, available as supplementary data at *Bioinformatics* online) and Neanderthal versus modern human DNA specifically (Section 3.5, Fig. 7, available as supplementary data at *Bioinformatics* online) within increasingly complex datasets. The exact mixtures used are to reflect datasets where the endogenous hominin DNA (Neanderthal in our examples) is the smallest component. In total 10 million reads were generated for each simulated dataset. We added double-stranded deamination for the Neanderthal reads under the Briggs model (Briggs *et al.* 2007) with an average length of 0.4 of single-stranded overhanging ends, 3% single-stranded nick frequency, 30% deaminated cytosine residuals in single-stranded DNA, and 1% deaminated in double-stranded DNA. We evaluated the performance by measuring two metrics: sensitivity defined as the number of identified reads that were correctly classified as “hominin” (Neanderthal or modern human) divided by the total number of hominin reads simulated, and precision defined as the number of identified reads that were truly hominin divided by the total number of identified reads.

Across simulations, we find that Centrifuge consistently had higher sensitivity than Kraken2, with minimal impacts on precision and lower memory requirements (Section 3.1, Figs 1 and 2, available as supplementary data at *Bioinformatics* online). Thus, we recommend Centrifuge as our default classifier. In addition, we find that performing classification before mapping reduces run times (Section 3.2, Fig. 3, available as supplementary data at *Bioinformatics* online) and using a classification cut-off at “primate” rather than “*Homo sapiens*” increases the sensitivity of identifying hominin reads (Section 3.3, Fig. 4, available as supplementary data at *Bioinformatics* online). Finally, we show that precision is impacted when using a smaller reference database because there is higher mis-classification of reads from non-hominin mammals as primate/hominin (Section 3.4, Figs 5 and 6, available as supplementary data at *Bioinformatics* online). These tests inform our recommendations for the *sedimix* workflow, which prioritizes maximizing the retention of accurately classified hominin sequences while maintaining computational tractability.

Using these recommendations, we then tested the sensitivity and specificity (with default settings) of *sedimix*. For the three simulated datasets described above, we find *sedimix* has both high sensitivity and specificity. Specifically, across the three simulated datasets, we achieved a sensitivity of 94.1%, 94.1%, and 94.7%, and a precision of 100%, 100%, 97.5%, respectively. The run time increased linearly with the number of input reads, and processing 100 million reads took 45 min on 32 Intel Xeon Gold 6330 CPUs. The memory requirement measured as the maximum resident set size (RSS) was 139 GB throughout the workflow, which should be manageable on most virtual computers.

After validating *sedimix’*s performance on simulated datasets, we explored its performance in real data by using the sequencing data published in two recent studies. The first by Gelabert *et al.* included ancient human DNA that was found in a 25,000 year old sediment sample (Gelabert *et al.* 2021). This dataset consists of a single fastq file with 522,997,582 raw sequencing reads. It required 4 h and 5 min for the analysis and reached a maximum memory usage of 141 GB. We identified 0.1% hominin DNA with ∼20% deamination rates on both ends, in close concordance with previous findings (Section 4, Table 4 and Fig. 8, available as supplementary data at *Bioinformatics* online). The second dataset from Essel *et al.* included ancient human touch DNA that was recovered from a cervid tooth (Essel *et al.* 2023). This dataset comprised five files with a total of 86,847,334 raw reads and took 2 h and 3 min for the analysis, with a peak memory usage of 139 GB. We identified ∼1% hominin sequences across three files, which is slightly higher than the published estimates though our estimated deamination rates are similar (Table 5, available as supplementary data at *Bioinformatics* online).

Together, these analyses showcase the reliability of *sedimix* for the recovery of hominin nuclear sequences from archaeological sediment samples, providing a robust and flexible tool for the future metagenomics applications.

## Supplementary Material

btag004_Supplementary_Data

## Data Availability

There are no new data associated with this article.
